# Data on metabolomic profiling of ovarian cancer patients' serum for potential diagnostic biomarkers

**DOI:** 10.1016/j.dib.2018.04.081

**Published:** 2018-04-30

**Authors:** Nejc Kozar, Kristi Kruusmaa, Marko Bitenc, Rosa Argamasilla, Antonio Adsuar, Nandu Goswami, Darja Arko, Iztok Takač

**Affiliations:** a Clinic of Gynaecology and Perinatology, University Medical Centre Maribor, Ljubljanska 5, 2000 Maribor, Slovenia; b Faculty of Medicine, University of Maribor, Taborska ulica 8, 2000 Maribor, Slovenia; c Faculty of Pharmacy, University of Ljubljana, Aškerčeva cesta 7, 1000 Ljubljana, Slovenia; d Universal Diagnostics, S.L. Centre of Research Technology and Innovation, University of Seville, Avenida Reina Mercedes s/n, 41012 Seville, Spain; e Institute of Physiology, Medical University of Graz, Harrachgasse 21/V, 8010 Graz, Austria

## Abstract

The data presented here are related to the research paper entitled “Metabolomic profiling suggests long chain ceramides and sphingomyelins as a possible diagnostic biomarker of epithelial ovarian cancer.” (Kozar et al., 2018) [1]. Metabolomic profiling was performed on 15 patients with ovarian cancer, 21 healthy controls and 21 patients with benign gynecological conditions. HPLC-TQ/MS was performed on all samples. PLS-DA was used for the first line classification of epithelial ovarian cancer and healthy control group based on metabolomic profiles. Random forest algorithm was used for building a prediction model based over most significant markers. Univariate analysis was performed on individual markers to determine their distinctive roles. Furthermore, markers were also evaluated for their biological significance in cancer progression.

**Specifications table**

**Table:** 

Subject area	*Gynecological oncology*
More specific subject area	*Ovarian cancer, ceramides, sphingomyelins, tumor markers*
Type of data	*Tables and Figures*
How data was acquired	*HPLC-TQ/MS*
Data format	*Analyzed*
Experimental factors	*Blood serum of ovarian cancer patients and two control groups*
Experimental features	*Metabolomic profiling of serum from ovarian cancer patients and control group*
Data source location	*Data was collected at University Medical Centre Maribor, Slovenia, while analysis was performed in Seville, Spain*
Data accessibility	*Data is provided within this article*

**Value of the data**

• Data about potential biomarkers in ovarian cancer patients are described in detail a form of univariate analysis with respective AUC information, that can be subsequently used by other researchers when selecting and analyzing potentially useful biomarkers for ovarian cancer diagnosis.
• Data from multivariate analysis is presented in detail for selected most important ovarian cancer biomarkers.
• Clinical data for every single patient included in the study is presented in a table providing means for studying ovarian cancer patient characteristics.
• Internal standards used in all analysis runs are described in table with exact composition.

## 1 Data

### 1.1 Data analysis

All data refers to the original reserach article entitled "Metabolomic profiling suggests long chain ceramides and sphingomyelins as a possible diagnostic biomarker of epithelial ovarian cancer." The data presented in Section 1.1 include univariate ROC curves analysis performed to evaluate the diagnostic power of all biomarkers when using them for differentiating between EOC patients and healthy control group. Analysis was performed based on data provided by HPLC-TQ/MS.

Using PLS-DA, biomarkers with best predictive value for separating between EOC patients and healthy control group were selected as shown in Table 1.

**Table 1:** Markers listed according to their discriminative power in PLS-DA analysis (VIP ≥2) of EOC and control samples.

**Biomarker**	**comp 1**	**comp 2**	**comp 3**	**global**
**Cer 34:1;2**	2.9	2.7	2.5	2.9
**Cer 40:1;2**	2.8	2.5	2.3	2.8
**Cer 42:1;2**	2.7	2.6	2.5	2.7
**DL-p-Hydroxyphenyllactic acid**	2.1	2.0	1.9	2.1
**Cer 44:1;2**	2.1	2.0	1.8	2.1
**PUFA 1**	2.1	2.0	1.8	2.1
**PUFA 4.1**	2.1	2.0	1.9	2.1
**SM 36:0;2**	2.1	1.9	1.8	2.1
**GLUTAMINE**	2.1	2.0	1.9	2.1
**SM 36:1;2**	2.0	1.8	1.7	2.0

Based on PLS-DA biomarker selection, multivariate analysis was performed alongside Monte Carlo cross validation (MCCV) to obtain classification/regression model with highest diagnostic power (Table 2).

**Table 2:** Markers found to be significantly contributing to disease classification according to multivariate analysis alongside MCCV.

*Biomarker*	Rank Freq.	VIP	CNTRL	OC
Cer 42:1;2	1	4.114932812	Low	High
Cer 40:1;2	1	3.990277021	Low	High
Cer 44:1;2	0.96	3.216162439	Low	High
Cer 34:1;2	0.92	2.755788672	Low	High
SM 36:0;2	0.78	2.218146547	Low	High
DL-p-Hydroxyphenyllactic acid	0.7	1.469915481	High	Low
PUFA 446	0.58	1.196797585	Low	High
SM 36:1;2	0.62	1.09797713	High	Low
LPA 16:0	0.54	1.023982275	Low	High

**Fig. 1:**
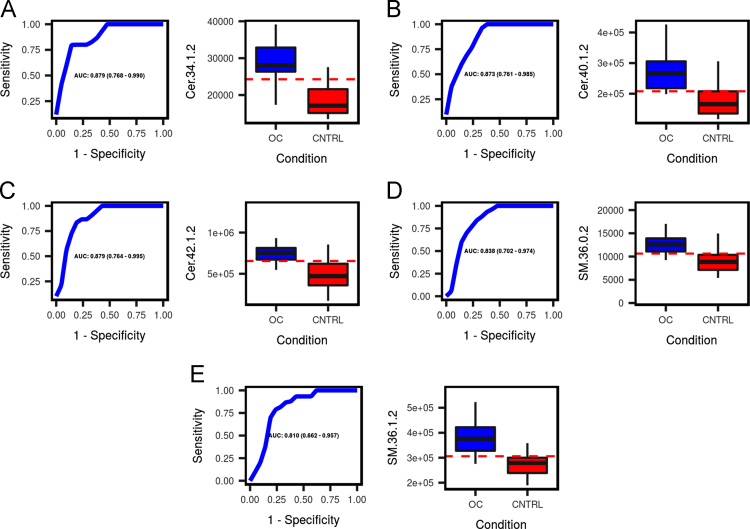
ROC curve and box-plot representation for 5 best performing markers when comparing EOC to pure control group.

Single marker prediction power and box-plot representation of each of the 5 markers were generated over univariate analysis of each individual marker. Predictions of single markers are presented in Fig. 1.

### 1.2 Patient's information

Section 1.2 presents clinical data from patients included in the study. Data was collected as a part of medical history and includes basic patient data such as age, BMI, smoking and menopause status along with all known medical diagnoses and current use of prescription or over the counter drugs.

### 1.3 Internal standards and quality control

Section 1.3 presents data about internal standards used in methanol solution over all analysis runs to ensure quality control.

## 2 Experimental design, materials and methods

### 2.1 Patient selection

All study subjects were recruited at the Clinic of Gynecology and Perinatology, University Medical Centre Maribor, which is one of the two Slovenian tertiary medical centers. It covers northeastern part of Slovenia and covers a population of approximately 800.000 inhabitants.

The study included 15 patients with diagnosed epithelial ovarian cancer, 21 patients without known gynecological condition and 21 patients with benign gynecological conditions.

Altogether 99 samples were collected for healthy control and benign disease cases and later on case control matching was performed based on age and BMI to produce the two 21 patients groups.

Clinical stages and histological classification based on the criteria of the International Federation of Gynecology and Obstetrics (FIGO) and the World Health Organization (WHO) were established in all cases. Ovarian cancer histopathology was established either with biopsy or post-surgically from tumor cancer tissues.

None of the patients were involved in any specific oncological treatment such as surgery, chemotherapy or radiotherapy prior to sample collection. Pre-treatment staging procedures included physical examination, laboratory workup, ultrasound and abdominal CT scanning and chest X-rays. In addition, bone scintigraphy, brain and thoracic CT imaging were performed as necessary.

Women included in the control group were mostly patients undergoing diagnostic evaluation for pelvic floor dysfunction. All patients were examined by a gynecologist prior to sample collection and the ultrasound examination was performed in every case. All patients and controls were received and treated at the Clinic of Gynecology and Perinatology, University Medical Centre Maribor, in the years 2014–2017. The study was approved by the national Ethics Committee (Approval no. 37/04/14) and all the patients gave their written informed consent for study participation.

The age of participants, menopausal status, additional diseases, use of prescription or over the counter drugs, smoking and alcohol use were registered at the time of sample collection while histopathological results were acquired additionally after the surgery or biopsy.

### 2.2 Sample collection

Serum samples from study subjects were collected prior to any specific treatment or surgery after minimum of 8 h fasting and avoiding smoking, alcohol and medication. Each participant was collected 5 ml of whole blood using BD Vacutainer Plus tubes with spray-coated silica. Serum extraction protocol was performed within the time period of 4 h from whole blood draw till freezing down serum. After centrifugation at 2000 *g* for 10 min at temperature of 4 °C serum samples were separated into four 500 µL portions and stored at −80 °C. After surgical treatment of patients and definitive histopathological results appropriate samples were taken into HPLC-TQ/MS analysis.

### 2.3 Metabolite and lipid extraction from serum

Serum samples were stored at −80 °C until thawed for analysis and were only thawed once. Frozen human serum samples were thawed at 4 °C using an ice bath and quality control (QC) pool was combined from all samples subjected to experiment and prepared alongside individual samples. Proteins were precipitated by mixing 30 µL of serum with 180 µL of Methanol at room temperature. The methanol solution contained 30 internal standards representing molecules over all analysis runs (Table 3). After homogenization of samples—using vortex for 2 min at maximum speed. The mixture was then centrifuged during 10 min at 12000 *g* and 10 °C. 160 µL supernatant was transferred to a new vial for analysis and mixed with 42 µL of water. All samples (prepared in duplicate), QC samples and blank standards were prepared as one analysis set and analyzed during one analysis run.

**Table 3:** Internal standards used in the analysis run.

**Name**	**ppm in MetOH**	**Name**	**ppm in MetOH**
>l-Tryptophan	0.8	2-d-Mannopyrasnosyl-l-tryptophan	0.03
Sebacic acid	0.019	Aspartylphenylalanine	0.045
>l-Tyrosine	2.1	N1,N12-Diacetylspermine	0.003973
1-Methyladenosine	0.019	Acetoacetate	3
Octanoylcarnitine (AC 8:0)	0.033	Azelaic acid	0.8
>l-Histidine	3.3	LPC 14:0	0.39
ADMA	0.039	Glycerophosphoethanolamine (GPE-2)	10.4
>l-Lysine	0.57	Choline	0.1
>l-Proline	1.5	Hippuric acid	0.3
Propionyl l-carnitine	0.019	Linoleic acid	7.5
>l-Pyrogutamic acid	0.33	N-acetylglycine	0.39
N-Acetylcytidine	0.1	Nicotineamide	0.012
Delta-Valerolactam	1.2	>l-Alanine	1.3
1,18-Octadecanedicarboxylic acid	0.026	3-Me-Glutaryl Carnitine	1
Sn-Glycero-3-phosphocholine	0.6		

### 2.4 Sample analysis

Transitions of 232 known metabolites selected based on literature and our previous unpublished results were targeted with 4 different analytical methods in dedicated AB Sciex TQ 4500 MD mass spectrometers that were coupled with Nexera X2 HPLC System from Shimadzu comprising a pump, auto sampler, controller and oven. QC samples and blank samples were analyzed after every 10 samples for evaluating stability of the system over long run and applying normalization for the samples.

### 2.5 LC–MS/MS

Four dedicated instruments for four different platforms using Shimadzu 20/30 AD 4500 coupled to Triple Quad/QTRAP (Sciex, Madrid, Spain) were used for all analytical samples.

#### 2.5.1 C18 polar analysis

An injection volume of 1 µL and a flow rate of 0.5 mL/min were used at column temperature of 40 °C. The mobile phases were aqueous solution (phase A) and acetonitrile (phase B) both complemented with 0.1%(v/v) formic acid. Separation of the metabolites was performed on a ACQUITY UPLC BEH C18 Column, 130 Å, 1.7 µm, 2.1 mm×50 mm attached to VanGuard Acquity UPLC BEH C18 1.7 μm for the column safety. The gradient method was as follows: 98% for 1 min and from 98% to 2% in 9 min (held 4 min). Multiquant Software was used to extract the areas of 67 known compound peaks.

#### 2.5.2 Lipid analysis

An injection volume of 1 µL and a flow rate of 0.4 mL/min were used at column temperature 65 °C. The mobile phases were:phase A- 40% water, 60% acetonitrile, 10 mM ammonium formiate, 0.1% formic acid and phase B- 10% acetonitrile, 85% isopropanol, 5% water, 10 mM ammonium formiate, 0.1% formic acid. Separation of the metabolites was performed on a ACQUITY UPLC BEH C18 Column, 130 Å, 1.7 µm, 2.1 mm×100 mm attached to VanGuard Acquity UPLC BEH C18 1.7 μm for the column safety. The gradient method was as follows: 85% of phase A for 1 min and the percentage of A changes to 70% in 2 min, then goes to 52% in 0.5 min, and goes to 18% in 8.5 min. Then A changes to 1% in 0.5 min where is held additional half minute. Then the percentage of A goes to initial conditions for column equilibration until 15 min. MultiQuant Software was used to extract the areas of 92 known compound peaks.

#### 2.5.3 Amide analysis

An injection volume of 1 μL and a flow rate of 0.5 mL/min were used at column temperature of 45 °C. The mobile phases were: phase A- 70% water, 30% acetonitrile, 10 mM ammonium formate, 0.1% formic acid and phase B- 1 95% acetonitrile, 5% water, 10 mM ammonium formate, 0.1% formic acid. Separation of the metabolites was performed on a ACQUITY UPLC BEH Amide Column, 130 Å, 1.7 µm, 2.1 mm×50 mm attached to VanGuard Acquity UPLC BEH C18 1.7 μm for the column safety. The gradient method was as follows: 80% for 2 min and from 80% to 20% in 2 min (held 4 min). MultiQuant Software was used to extract the areas of 56 known compound peaks.

#### 2.5.4 FIA (flow injection analysis)

An injection volume of 2 μL and a flow rate of 0.36 mL/min were used. The mobile phase was 100% Isocratic with run-time 1 min. MultiQuant Software was used to extract the areas of 17 known compound peaks.

MultiQuant software was used for evaluating the integrity of all the peaks generated via analysis and for generating area intensity files.

### 2.6 Statistical analysis

Partial least squares Discriminant Analysis (PLS-DA) statistical method was used to find the best linear predictor of potential markers based on the dependent variables X (sample readings). Before PLS-DA, data were batch normalized as dividing each variable of each batch by the square root of the mean of the squares of all original values of that batch. Finally the dataset was log transformed and scaled by using pareto scaling method (reduce relative importance of large values, partially preserve data structure).

Univariate ROC curves analysis was performed to evaluate the diagnostic power of all elements and ratios by using ROCCET package in r. Before univariate ROC curves analysis data was batch normalized by dividing each variable of each batch by the square root of the mean of the squares of all original values of that batch. Finally the dataset was log transformed and scaled by pareto scaling.

Final Data Analysis was performed by random forest based in-house customized algorithm. For significant marker selection, the SBS (sequential backward selection) was used. SBS relies on a random forest classification algorithm. Using the OOB error as minimization criterion, carry out variable elimination from random forest, by successively eliminating the least important variables (with importance as returned from random forest). Monte Carlo cross validation (MCCV, developed using in-house scripts) was then conducted over significant markers. For each iteration of MCCV, the n samples were first randomly split into two parts, the training set (Xtrain, ytrain) and testing set (Xtest, ytest). The MCCV procedure was repeated *N* times (i.e., *N*=50), and the average and distribution of predictive performance was calculated (i.e., AUC using ŷtest). Composite average ROC curves were constructed to summarize overall classification accuracy (R package: ROCR).

## Appendix A Supplementary material

Table 1. Univariate analysis data.

Table 4. Detailed EOC group clinical data

Table 5. Detailed healthy control group clinical data.

Table 6. Detailed benign disease group clinical data.
