# Supplementary material for: Data on metabolomic profiling of ovarian cancer patients' serum for potential diagnostic biomarkers
Source: Data Brief. 2018 Apr 30;18:1825–31. doi: 10.1016/j.dib.2018.04.081 (PMC5998211; doi:10.1016/j.dib.2018.04.081)
Supplement: Supplementary file 1 — Supplementary material [file mmc1.pdf]

Declaration of interests

KK, MB, RA, AA are employees of a company Universal DX that may in a long term profit from commercially available diagnostic test developed partially on datasets and results published in this study.
